# Impressions of Osteopathic Neurosurgeon on Preparedness for Practice: Survey Results from the American College of Osteopathic Surgeons

**DOI:** 10.7759/cureus.5757

**Published:** 2019-09-25

**Authors:** James Brazdzionis, Harjyot Toor, Tye Patchana, James G Wiginton, Raed Sweiss, Margaret Rose Wacker, Vladimir Cortez, Dan E Miulli

**Affiliations:** 1 Neurosurgery, Riverside University Health System Medical Center, Moreno Valley, USA; 2 Neurosurgery, Arrowhead Regional Medical Center, Colton, USA; 3 Neurosurgery, Desert Regional Medical Center, Palm Springs, USA

**Keywords:** neurosurgery, medical education, liability, single accreditation, osteopathic, training, perception, survey, preparedness, aoa

## Abstract

Introduction

Neurosurgeons trained in the US are rigorously educated on the surgical management of neurosurgical conditions. These neurosurgeons have been trained through one of two avenues: the Accreditation Council for Graduate Medical Education (ACGME) or the American Osteopathic Association (AOA). With the formation of a single accreditation system from the AOA and ACGME accrediting bodies and significant changes introduced in the training of neurosurgeons from both bodies, we sought to identify common practice parameters and perceptions of preparedness of AOA-trained neurosurgeons.

Methods

A survey was conducted through the neurosurgery section of the American College of Osteopathic Surgeons (ACOS), requesting responses from attending neurosurgeons who completed AOA neurosurgery residency. Responses were obtained through an anonymous, web-based system using single-select multiple-choice questions.

Results

In total, 52 neurosurgeons participated in the survey. The majority of the 52 respondents practiced in non-academic settings in urban areas and were exposed to a wide variety of practice environments in terms of case volume and clinical responsibilities. Significantly, 96.15% of the respondents said they felt adequately prepared for neurosurgical practice after their AOA training.

Conclusion

Overall, this study highlights both the similarities and variances in practices of osteopathic neurosurgeons. The majority of the participants feel that their training has appropriately prepared them for practice and they are skilled surgeons capable of caring for the safety and well-being of numerous patients in a variety of settings. Most of them practice primarily in private-practice settings at urban centers. Overall, osteopathic neurosurgeons trained in AOA programs report that their training has equipped them well for careers in neurosurgery.

## Introduction

Neurosurgical training in the US is a well-developed and structured academic milieu to train surgeons at providing optimal care for surgical neurological conditions. Historically, in the United States, there have been two avenues for training and board certification for neurosurgeons: neurosurgical residencies at the Accreditation Council for Graduate Medical Education (ACGME) and the American Osteopathic Association (AOA). In light of the planned formation of a single accreditation from the AOA and ACGME residencies and the increasingly intertwined practices of the osteopathic- and allopathic-trained neurosurgeons, we wished to conduct a survey to identify common factors in practice as well as perceptions of preparedness for AOA-trained neurosurgeons. Single accreditation will likely bring changes to the way osteopathic neurosurgery residencies have traditionally operated [1]. Neurosurgeons have been practicing for many years after training through both the accrediting bodies, and support for investigations of preparedness for neurosurgeons have been promoted by multiple neurosurgical organizations. However, there is a dearth of significant data about these aspects. The only identified study investigating preparedness among ACGME-trained neurosurgeons is by Mazzola et al. [2]. We believe ours is the first paper to investigate preparedness to practice through osteopathic training in neurosurgeons.

## Materials and methods

Members of the neurosurgery division of American College of Osteopathic Surgeons (ACOS) who had completed osteopathic neurosurgical training in an AOA-accredited neurosurgical residency were invited to complete an anonymous survey. Invitations for the survey were sent out via email to the aforementioned neurosurgeons on behalf of ACOS. One reminder email was sent out as well to each of the recipients to encourage participation. Additionally, the survey was advertised on the ACOS website. The survey was completed through an anonymous, online platform and remained open for two months. The survey questions were multiple choice with single-selection answers. Physicians were able to defer answering questions if they so chose.

## Results

**Figure 1:**
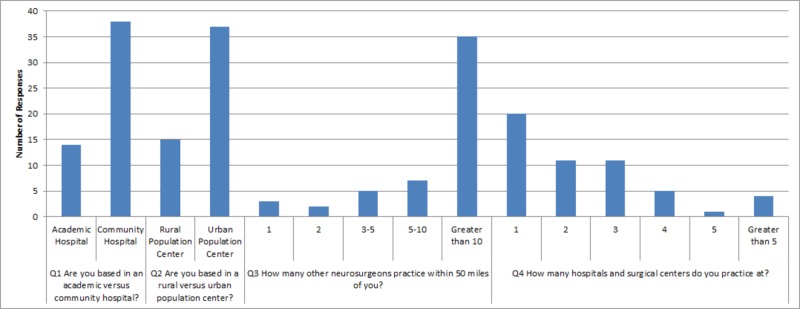
Graphical representation of survey responses for questions one through four Q: question

**Figure 2:**
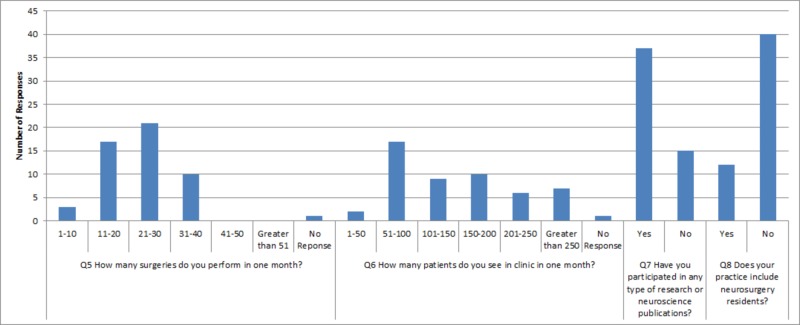
Graphical representation of survey responses for questions five through eight Q: question

**Figure 3:**
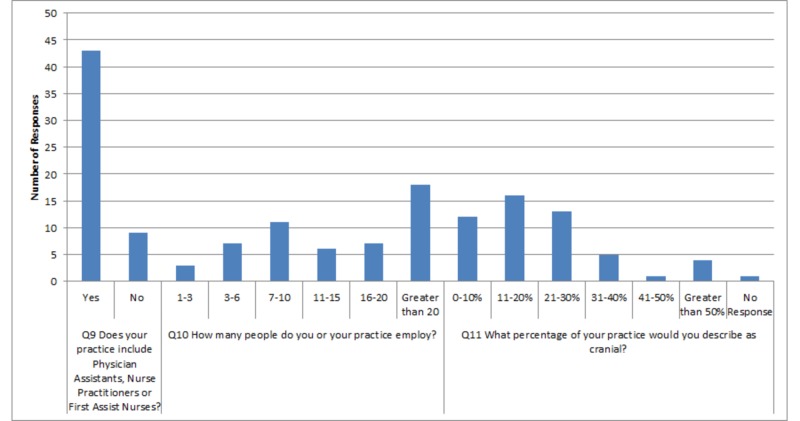
Graphical representation of survey responses for questions nine through eleven Q: question

**Figure 4:**
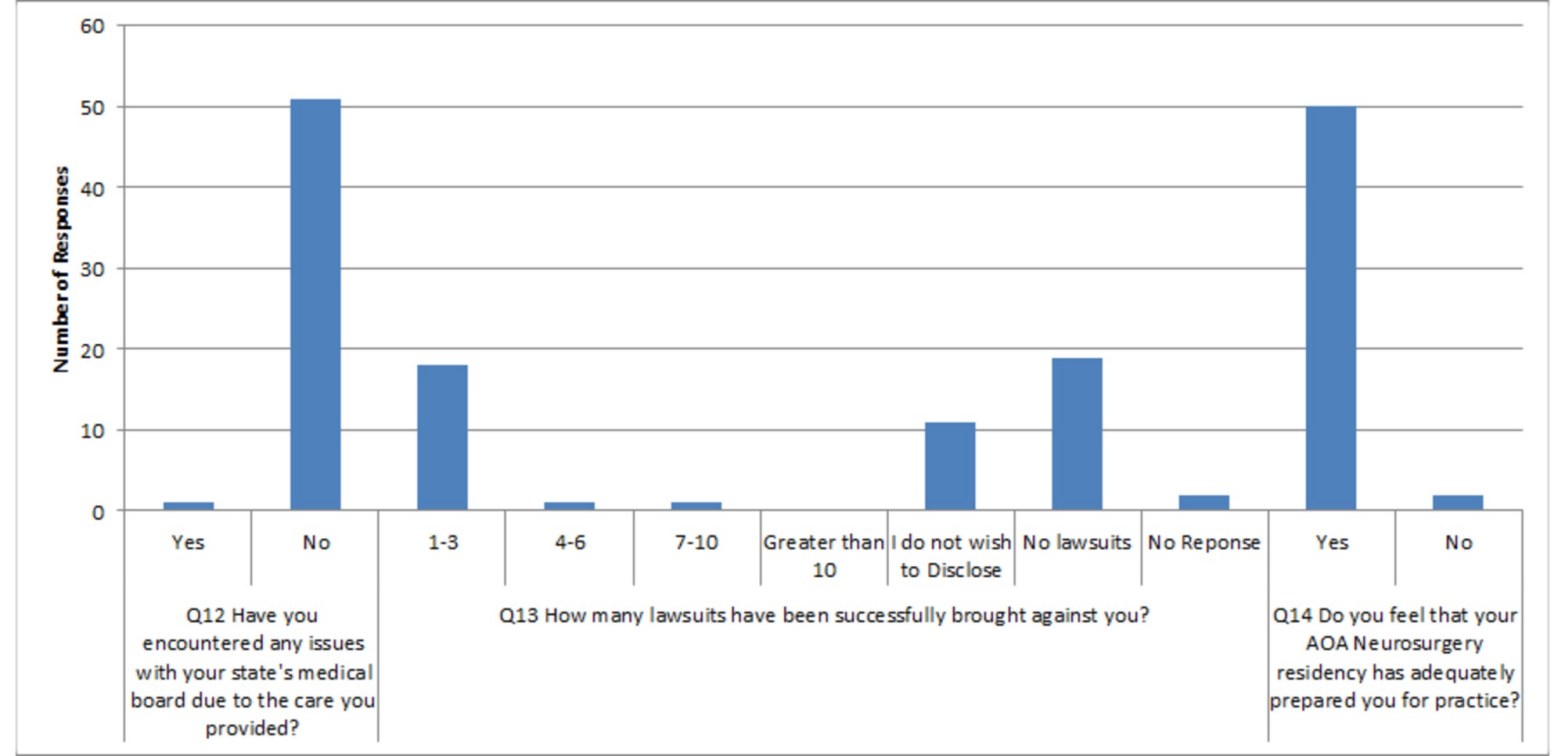
Graphical representation of survey responses for questions twelve through fourteen Q: question

Of the 195 Osteopathic neurosurgeons who are ACOS members, 52 responded to the anonymous questionnaire. All of them responded to all of the questions with the exception of questions 5, 6, 11, and 13. Of these skipped questions, there was one skipped response for questions 5, 6, and 11 and two skipped responses for question 13. Responses to questions are displayed in a graphical format below (Figures 1-4) and in table format in the Appendix (Table 1).

## Discussion

This survey evaluated impressions of neurosurgical training from AOA-board certified neurosurgeons trained in AOA residencies in the US. Overall, these individuals tend to practice in community settings in urban population centers (Figure 1). The majority practice at more than one surgical center or hospital. The most common response of these neurosurgeons indicates that they usually perform anywhere from 21 to 30 surgeries per month, but occasionally tend to range anywhere from 11 to 40 surgeries per month. Clinically, they see a variable number of patients in clinics, with the most common response stating anywhere from 51 to 100 per month. Many are academically oriented with 71.15% participating in research; however, they do not tend to have neurosurgical-residency involvement. The majority (82.69%) employ mid-level providers or first-assist nurses. Practice sizes are varied and diverse. However, 80.39% perform 30% or less cranial neurosurgery in their practice (Table 1).

AOA-trained neurosurgeons surveyed are safe practitioners with only one responder having encountered any issue with their state licensing board due to issue with the care provided (Table 1). The most frequent response in regards to lawsuits was "no successful lawsuits being levied against the neurosurgeon" (38%). However, 11 neurosurgeons (22%) specifically stated that they did not wish to disclose anything regarding this, and another two responders skipped this question altogether. This is important to note as a recent study demonstrated that approximately 19.1% of neurosurgeons face lawsuits each year [3,4]. Overall, neurosurgeons' perception of their training was positive with 96.15% (50 out of 52) of the responders believing their training adequately prepared them for neurosurgical practice. 

A similar article surveying ACGME-trained neurosurgeons has been published, which reported overwhelmingly positive responses about their training [2]. In terms of practice, these neurosurgeons practice more frequently in academic environments (43% of the responses) than osteopathic neurosurgeons (26.92% of the responses). However, it is important to note that there was a very limited response rate in this study, with only 30% of the 775 surveyed neurosurgeons participating. In terms of overall perceptions of training, 98.65% (220 of the 223) responders believe they were adequately or excessively prepared in general neurosurgery, while only 3 believe they were not adequately trained for general neurosurgical practice. 

In an environment with increasing integration in residency training, it is becoming more important to identify these training perceptions to ensure that appropriately trained neurological surgeons are able to fully develop and advance their careers. This is occurring at a time when there is a lack of consensus over the enacted ACGME work-hour restrictions [5]. Our data on AOA-trained neurosurgeons compare favorably with ACGME-trained neurosurgeons pertaining to their perceptions of the training they underwent. 

There are some limitations to this study. As this study was designed as a survey, responses were voluntary and not all osteopathic neurosurgeons completed the survey. Other limitations are the kind intrinsic to any survey as individuals who have strong positive or negative opinions are more likely to complete the survey when compared to those who do not hold strong views. Furthermore, surveys, in general, are thought to have an intrinsic bias towards positive answers. Although responders were assured of anonymity, individuals may still have generally provided responses that skewed toward more positive aspects for fear of lack of full anonymity.

## Conclusions

This study attempted to highlight both the similarities and variances in practices of osteopathic neurosurgeons. The majority of the participants feel that their training has appropriately prepared them for practice and they are safe and effective surgeons capable of caring for numerous patients. They practice primarily in private settings at urban centers. Many also practice in a variety of settings including academic and rural centers. Overall, osteopathic neurosurgeons are satisfied with their training and are able to safely and effectively practice in a variety of environments and settings. They feel that their training has equipped them well for careers in neurosurgery.

## Appendices

**Table 1:** Tabulation for responses from surveyed osteopathic neurosurgeons. Calculated percentage of responses do not include those of people who did not respond to the question Q: question; AOA: American Osteopathic Association

Questions	Number of responses	Percentage of responses
Q1. Are you based in an academic versus community hospital?		
Academic Hospital	14	26.92%
Community Hospital	38	73.08%
Q2. Are you based in a rural versus urban population center?		
Rural Population Center	15	28.85%
Urban Population Center	37	71.15%
Q3. How many other neurosurgeons practice within 50 miles of you?		
1	3	5.77%
2	2	3.85%
3-5	5	9.62%
5-10	7	13.46%
Greater than 10	35	67.31%
Q4. How many hospitals and surgical centers do you practice at?		
1	20	38.46%
2	11	21.15%
3	11	21.15%
4	5	9.62%
5	1	1.92%
Greater than 5	4	7.69%
Q5. How many surgeries do you perform in one month?		
1-10	3	5.88%
11-20	17	33.33%
21-30	21	41.18%
31-40	10	19.61%
41-50	0	0.00%
Greater than 51	0	0.00%
No Response	1	
Q6. How many patients do you see in clinic in one month?		
1-50	2	3.92%
51-100	17	33.33%
101-150	9	17.65%
150-200	10	19.61%
201-250	6	11.76%
Greater than 250	7	13.73%
No Response	1	
Q7. Have you participated in any type of research or neuroscience publications?		
Yes	37	71.15%
No	15	28.85%
Q8. Does your practice include neurosurgery residents?		
Yes	12	23.08%
No	40	76.92%
Q9. Does your practice include physician assistants, nurse practitioners, or first-assist nurses?		
Yes	43	82.69%
No	9	17.31%
Q10. How many people do you or your practice employ?		
1-3	3	5.77%
3-6	7	13.46%
7-10	11	21.15%
11-15	6	11.54%
16-20	7	13.46%
Greater than 20	18	34.62%
Q11. What percentage of your practice would you describe as cranial?		
0-10%	12	23.53%
11-20%	16	31.37%
21-30%	13	25.49%
31-40%	5	9.80%
41-50%	1	1.96%
Greater than 50%	4	7.84%
No Response	1	
Q12. Have you encountered any issues with your state's medical board due to the care you provided?		
Yes	1	1.92%
No	51	98.08%
Q13. How many lawsuits have been successfully brought against you?		
1-3	18	36.00%
4-6	1	2.00%
7-10	1	2.00%
Greater than 10	0	0.00%
I do not wish to Disclose	11	22.00%
No lawsuits	19	38.00%
No Response	2	
Q14. Do you feel that your AOA Neurosurgery residency has adequately prepared you for practice?		
Yes	50	96.15%
No	2	3.85%
